# The influence of duloxetine on detrusor overactivity in rats with depression induced by 13-*cis*-retinoic acid

**DOI:** 10.1007/s00192-017-3424-2

**Published:** 2017-07-31

**Authors:** Andrzej Wróbel, Ewa Rechberger, Tomasz Rechberger

**Affiliations:** Second Department of Gynecology, Medical University of Lublin, Jaczewskiego 8, PL 20-090 Lublin, Poland

**Keywords:** Duloxetine, Detrusor overactivity, Depression, Cystometry, Rats

## Abstract

**Introduction and hypothesis:**

The aim of this study was to assess the efficacy of duloxetine in an animal model of detrusor overactivity induced by depression.

**Methods:**

After 6 weeks of 13-*cis*-retinoic acid administration at a dose of 1 mg/kg/day, rats were given duloxetine at a dose of 1 mg/kg. This was followed by conscious cystometry, a forced swim test, and locomotor activity measurement. The levels of corticotropin-releasing factor (CRF) in the hypothalamus, amygdala and plasma were also determined.

**Results:**

Duloxetine treatment led to a reduction in detrusor overactivity symptoms induced by the retinoid. Decreases were observed in cystometric parameters including the detrusor overactivity index, and the amplitude and frequency of nonvoiding contractions, while increases were seen in bladder compliance and the volume threshold to elicit nonvoiding contractions. No statistically significant differences were found in basal pressure, threshold pressure, micturition voiding pressure, postvoid residual , volume threshold, voiding efficiency, intercontraction interval, bladder contraction duration or relaxation time. Duloxetine also reduced the immobility time to that observed in control animals, while it did not affect locomotor activity. Its effects also included lowering of the CRF levels in the hypothalamus, amygdala and plasma, which increased following the prior administration of the retinoid. The plasma level of 13-*cis*-retinoic acid in rats corresponded to the levels found in humans.

**Conclusions:**

This is the first study showing the efficacy of duloxetine in an animal model of detrusor overactivity induced by depression. Further studies in patients with detrusor overactivity and coexisting depression are warranted to confirm these experimental results.

## Introduction

Patients with various clinical types of urinary incontinence are frequently diagnosed with mental disorders including depression. Although the putative link between such urinary tract malfunction was first described in 1964 [1], probably the first to speculate on common neurological pathways for depression and detrusor overactivity (DO) leading to both wet and dry overactive bladder (OAB) were Steers and Lee [2]. Indeed some data suggest that depression might be associated with OAB syndrome [3], but very few studies have focused on OAB patients with clinically confirmed psychiatric disorders [4, 5]. Epidemiological studies have indicated that such a link may exist. It has been demonstrated that patients with OAB have significantly worse results on the Centre of Epidemiological Studies Depression (CES-D) and quality of sleep scales [6], as well as on the Short Form Health Survey (SF-36) quality of life scale. A positive correlation has been found between depression and the development of the first symptoms of OAB. It has been suggested that depression, as assessed with the Geriatric Depression scale, is a risk factor for OAB [7]. Of patients diagnosed with depression, 13% also had symptoms of stress urinary incontinence (SUI) and 42% had symptoms of OAB or mixed urinary incontinence (MUI), and as many as 60% of patients with idiopathic urge incontinence had a history of depression or a Beck’s Depression Inventory score of >12.

Corticotropin-releasing factor (CRF) is considered a primary initiator of changes leading to depression. Its level increases especially in the paraventricular nucleus of the hypothalamus, the central nucleus of the amygdala, and Barrington’s nucleus, the pontine micturition centre. It displays strong psychotropic properties, predominantly as depressive reactions. Preclinical studies have revealed that CRF induces depressive reactions by increasing the immobility time in forced swim tests. The CRF_1_ receptor antagonists show, in turn, antidepressant properties [8]. In forced swim tests the reduction in passive (immobility) behaviour is interpreted as an antidepressant-like effect [9]. Clinical trials have confirmed that patients with depression have significantly increased CRF levels in their cerebrospinal fluid. Post-mortem examinations have also revealed elevated CRF concentrations in the hypothalamus and an increase in the number of CRF-releasing neurons located in the paraventricular nucleus of the hypothalamus.

The effects of CRF are mediated by CRF_1_ and CRF_2_ receptors. Brain regions characterized by high concentrations of these receptors, such as Barrington’s nucleus in the brainstem amygdala, the prefrontal cortex and the hippocampus, play an important role, not only in the aetiopathogenesis of depression but also in voiding control. The neurons of Barrington’s nucleus, which project to the bladder motoneurons, are considered the switch that initiates the descending limb of the micturition reflex. There is some evidence suggesting that high CRF expression of the neurons of Barrington’s nucleus is an important factor regulating bladder detrusor function. It has been found that CRF lowers the threshold of the afferent impulsation of the voiding reflex and increases the contractile activity of the detrusor muscle, whose levels increase in response to bladder distension. In vivo cystometric tests have confirmed the excitatory effects of CRF on micturition [10]. CRF_1_-selective antagonists have been found to have a reducing effect on DO. CRF has an effect on the micturition reflex, including at the level of the spinal cord. It has been shown that capsaicin, by selectively destroying afferent C-fibres, induces a decrease in CRF expression, confirming that this neurohormone is vital in the conduction of afferent impulsation from the bladder [11].

Duloxetine is a dual reuptake inhibitor of serotonin and norepinephrine (SNRI) without considerable affinity for these neurotransmitter receptors, and has been approved for the treatment of major depression and SUI. Noradrenaline and serotonin terminals are widespread in brain structures responsible for the body’s response to stress, especially in the limbic system. Deficiency in these neurotransmitters induces the development of depression, which is an indication for the administration of SNRIs such as duloxetine in patients with clinical depression [12].

It has been demonstrated that duloxetine increases bladder capacity by increasing urethral sphincter muscle activity without affecting the micturition phase [13]. By inducing an increase in serotonin and norepinephrine concentrations, it modulates the activity of the motoneurons of Onuf’s nucleus, and in this way prevents accidental bladder voiding. Only a few sources suggest that duloxetine can inhibit DO development and modulate sensory processing, which would justify its use in OAB treatment [14–16]; however, the treatment of patients with OAB with this SNRI has not yet been indicated. At present there are no data on the use of duloxetine in the treatment of patients with depression and accompanying OAB symptoms.

These observations taken together were the basis for the experiments presented here, which had the aim of assessing the effectiveness of duloxetine in an animal model of DO induced by depression [17]. Furthermore, the effects of this SNRI on CRF levels in plasma and brain structures that play a crucial role in both the aetiopathogenesis of depression and the occurrence of OAB were also assessed.

## Materials and methods

### Animals

All procedures were conducted in accordance with the European Communities Council Directive of 22 September 2010 (2010/63/EU) and were approved by the Ethics Committee of the Medical University of Lublin.

A total of 60 female Wistar rats were used and randomly assigned to one of the following four treatment groups (15 animals per group):

1. Control
2. Duloxetine (1 mg/kg)
3. 13-*cis*-Retinoic acid (13-*cis*-RA; 1 mg/kg/day)
4. 13-*cis*-RA plus duloxetine

Rats were experimentally naive and tested once. The animals were weighed at intervals of 1 week until completion of the experiment.

### Drugs

The following drugs were used:

- 13-*cis*-RA (Tocris Bioscience, Bristol, UK) was dissolved in the dark in a mixture of dimethyl sulphoxide and physiological saline at a ratio of 1:1 just before administration. The solution was injected intraperitoneally at a dose of 1 mg/kg/day for 6 weeks at a volume of 1 ml/kg body weight.
- Duloxetine hydrochloride (Eli Lilly, Indianapolis, IN) was dissolved in distilled water and administered intravenously as a single dose of 1 mg/kg at a volume of 1 ml/kg body weight.

The doses of the administered agents were taken from the literature and were confirmed/adjusted in our laboratory in preliminary experiments.

### Study design

After 6 weeks of 13-*cis*-RA administration, cystometric studies were performed to assess the effect of 13-*cis*-RA on the micturition cycle and to examine the influence of duloxetine on the cystometric parameters modified by 13-*cis*-RA treatment. They were immediately followed by behavioural tests. Blood samples were then collected and the hypothalamus and amygdala were isolated to determine the levels of 13-*cis*-RA and CRF.

### Surgical procedures

The surgical procedures have been previously described in detail [18]. In brief, the abdominal wall was opened with a vertical midline incision of approximately 10 mm. A double-lumen catheter was inserted through the apex of the bladder dome and fixed with a 6-0 suture. In the same session the right femoral vein was catheterized via an inguinal approach.

### Conscious cystometry

Cystometric investigations were performed 3 days after the surgical procedures and after 6 weeks of 13-*cis*-RA treatment. The bladder catheter was connected via a three-way stopcock to a pressure transducer (FT03) and to a microinjection pump (CMA 100). Cystometry was performed by slowly filling the bladder with physiological saline at a constant rate 0.05 ml/min to elicit repetitive voiding. Micturition volumes were measured by means of a fluid collector attached to a force displacement transducer (FT03C). The measurements in each animal represent the average of five bladder micturition cycles after obtaining repetitive voiding. The mean values from all animals in each condition were averaged to create pooled data for each condition.

The following cystometric parameters were recorded: basal pressure (BP, cm H_2_O), threshold pressure (TP, cm H_2_O), micturition voiding pressure (MVP, cm H_2_O), voided volume (VV, ml), postvoid residual (PVR, ml), volume threshold (VT, ml), voiding efficiency (VE, %), intercontraction interval (ICI, s), bladder contraction duration (BCD, s), relaxation time (RT, s), bladder compliance (BC, ml/cm H_2_O), and DO index (DOI, cm H_2_O/ml; calculated as the sum of the amplitudes of all detrusor contractions during the filling phase divided by the functional bladder capacity [17–20]), amplitude of nonvoiding contractions (ANVC, cm H_2_O), frequency of nonvoiding contractions (FNVC, times/filling phase), and volume threshold to elicit NVC (VTNVC, %) [19].

### Locomotor activity

The locomotor activity of animals was assessed using a Digiscan apparatus. Horizontal activity was defined as the total number of infrared light beam interruptions during 1 h of measurement.

### Forced swim test

The immobility time was measured. The rats were considered to be immobile when they remained floating passively, performing slow motion movements to keep their head above the water.

### Determining the plasma level of 13-*cis*-retinoic acid

The plasma levels of 13-*cis*-RA were measured using reversed-phase high-performance liquid chromatography. The absorbance of the samples was identified at 354 nm and 13-*cis*-RA concentrations were determined by comparison with a standard curve.

### Determining the hypothalamus, amygdala and plasma levels of CRF

Using the stereotactic atlas of the rat’s brain and the bregma as the point of reference, the hypothalamus and amygdala were isolated. CRF concentrations were determined using a high-sensitivity commercial immunoenzymatic assay (Alpco, Salem, NH, USA) in line with the manufacturer’s instructions.

### Statistical analysis

The data obtained were evaluated by one-way analysis of variance followed by Tukey’s post hoc test (Statistica, v. 10). All results are presented as the means ± standard error of the mean (SEM). Values of *p* <0.05 were considered to indicate statistically significant differences.

## Results

The body weights of the rats measured weekly did not show any significant differences between the animal groups examined throughout the entire experiment. Daily monitoring of the animals confirmed that they did not manifest any signs of distress as a result of the repeated injections.

### Cystometric study

**Fig. 1:**
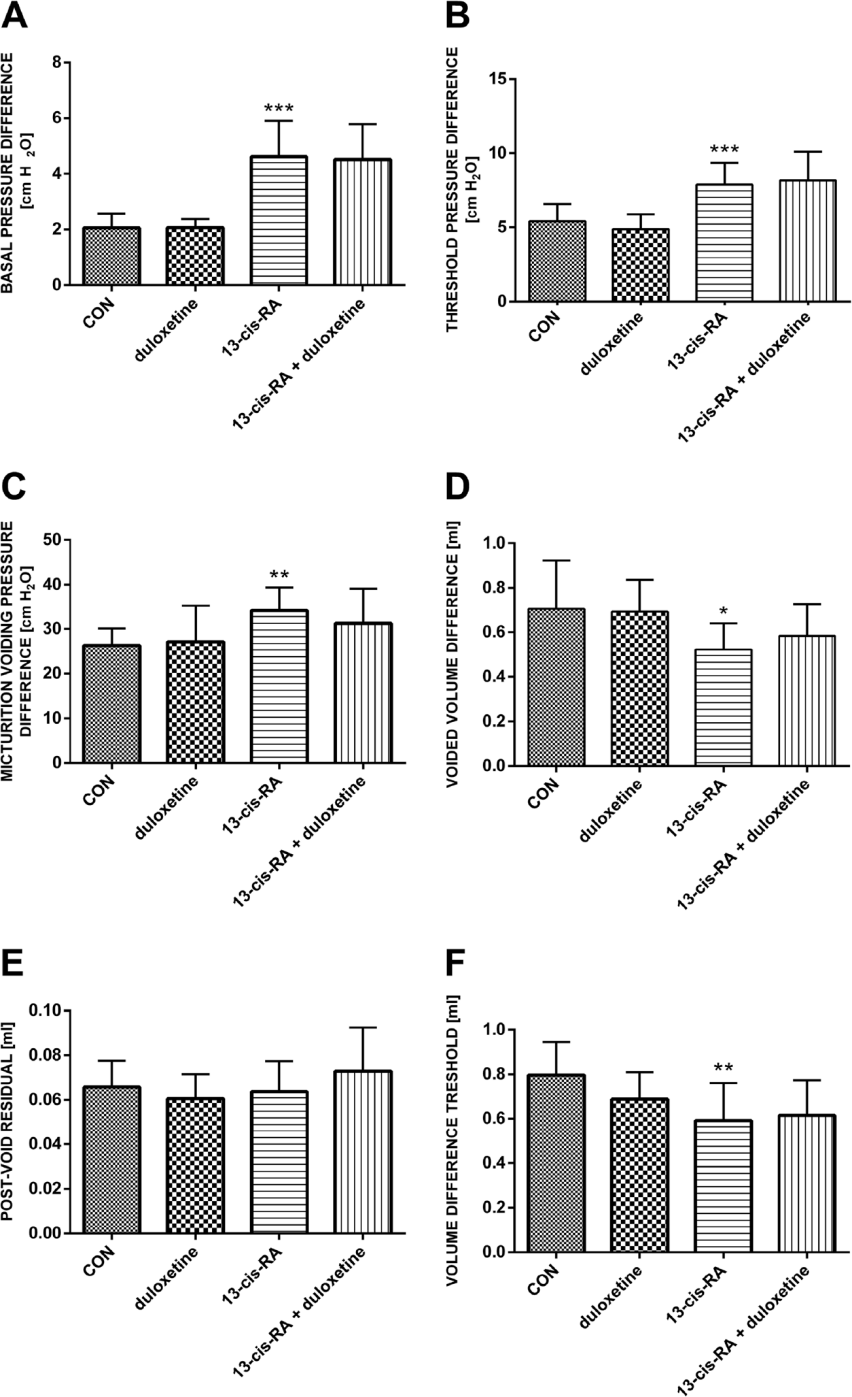
Mean values of cystometric parameters (basal pressure, threshold pressure, micturition voiding pressure, voided volume, postvoid residual, and volume threshold) characteristic of detrusor overactivity induced by treatment with 13-*cis*-RA in the different treatment groups showing the effects of treatment with duloxetine (*CON* control group). **a** Basal pressure (***p < 0.001, CON vs. 13-*cis*-RA). **b** Threshold pressure (***p < 0.001, CON vs. 13-*cis*-RA). **c** Micturition voiding pressure (**p < 0.01, CON vs. 13-*cis*-RA). **d** Voided volume (*p < 0.05, CON vs. 13-*cis*-RA). **e** Postvoid residual. **f** Volume threshold (**p < 0.01, CON vs. 13-*cis*-RA). All results are presented as means ± SEM (*n* = 15 rats per group). The data obtained were evaluated using one-way analysis of variance followed by Tukey’s post hoc test. *p < 0.05, **p < 0.01, ***p < 0.001

**Fig. 2:**
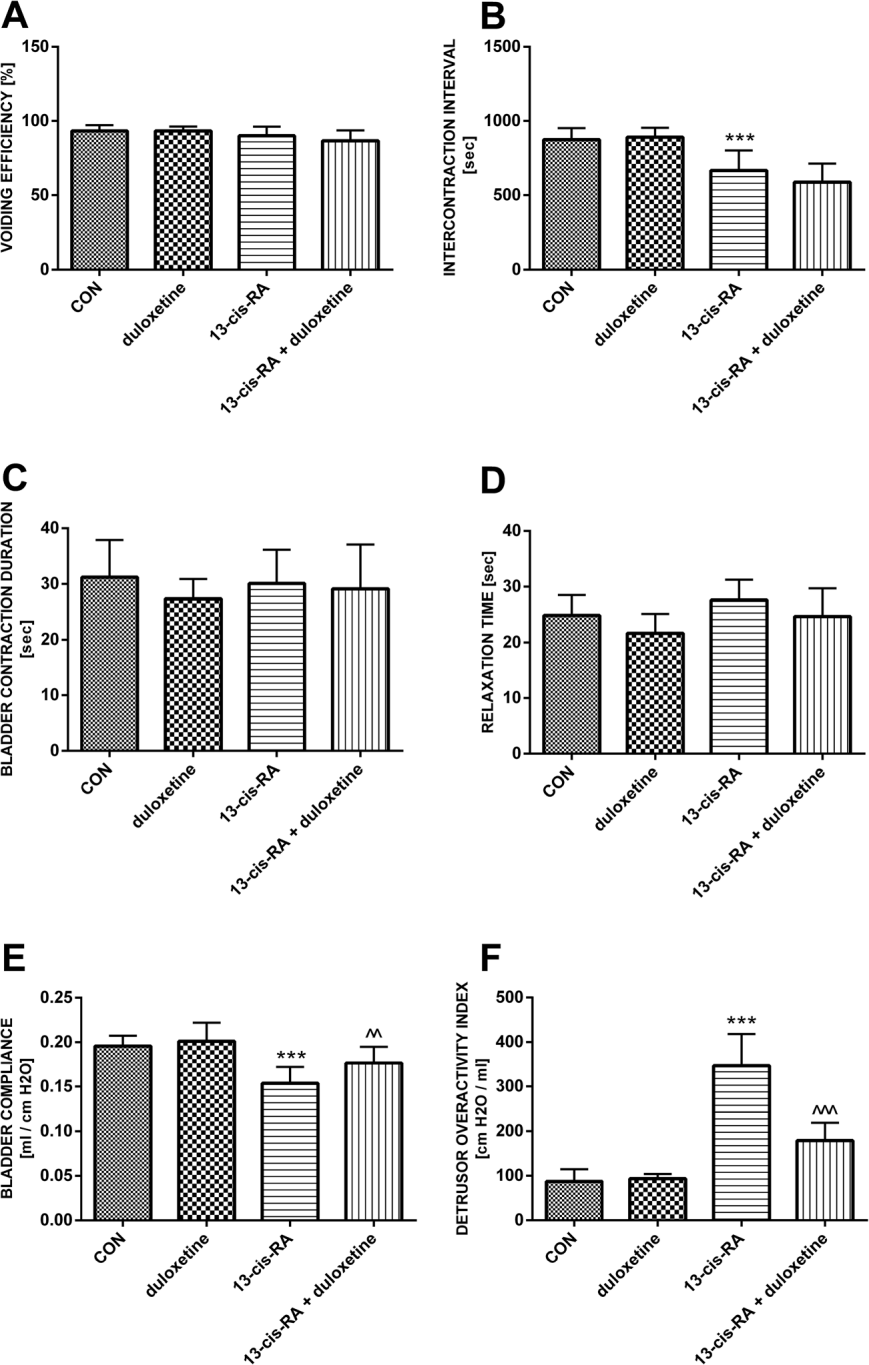
Mean values of cystometric parameters (voiding efficiency, intercontraction interval, bladder contraction duration, relaxation time, bladder compliance, and detrusor overactivity index) characteristic of detrusor overactivity induced by treatment with 13-*cis*-RA in the different treatment groups showing the effects of treatment with duloxetine (*CON* control group). **a** Voiding efficiency. **b** Intercontraction interval (***p < 0.001, CON vs. 13-*cis*-RA). **c** Bladder contraction duration. **d** Relaxation time. **e** Bladder compliance (***p < 0.001, CON vs. 13-*cis*-RA; ^^*p* < 0.01, 13-*cis*-RA vs. 13-*cis*-RA + duloxetine. **f** Detrusor overactivity index (***p < 0.001, CON vs. 13-*cis*-RA; ^^^*p* < 0.001, 13-*cis*-RA vs. 13-*cis*-RA + duloxetine). All results are presented as means ± SEM (*n* = 15 rats per group). The data obtained were evaluated using one-way analysis of variance followed by Tukey’s post hoc test. ^^*p* < 0.01, ^^^*p* < 0.001, ***p < 0.001

**Fig. 3:**
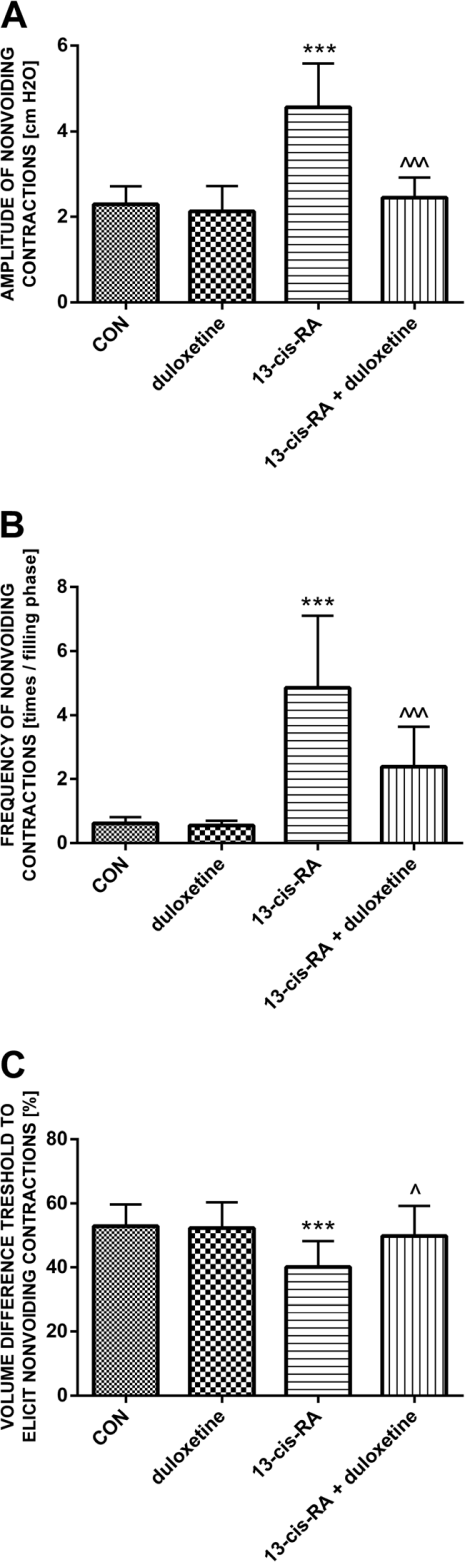
Mean values of cystometric parameters (amplitude of nonvoiding contractions, frequency of nonvoiding contractions, and volume threshold to elicit nonvoiding contractions) characteristic of detrusor overactivity induced by treatment with 13-*cis*-RA in the different treatment groups showing the effects of treatment with duloxetine (*CON* control group). **a** Amplitude of nonvoiding contractions (***p < 0.001, CON vs. 13-*cis*-RA; ^^^*p* < 0.001, 13-*cis*-RA vs. 13-*cis*-RA + duloxetine). **b** Frequency of nonvoiding contractions (***p < 0.001, CON vs. 13-*cis*-RA; ^^^*p* < 0.001, 13-*cis*-RA vs. 13-*cis*-RA + duloxetine). **c** Volume threshold to elicit nonvoiding contractions (***p < 0.001, CON vs. 13-*cis*-RA; ^*p* < 0.05, 13-*cis*-RA vs. 13-*cis*-RA + duloxetine). All results are presented as means ± SEM (*n* = 15 rats per group). The data obtained were evaluated using one-way analysis of variance followed by Tukey’s post hoc test. ^*p* < 0.05, ^^^*p* < 0.001, ***p < 0.001

Rats treated with a single dose of duloxetine did not show any significant differences in cystometric parameters compared with the control group. Treatment with 13-*cis*-RA led to changes in cystometric parameters characteristic of DO. Increases were observed in BP, TP, MVP, DOI, ANVC and FNVC, while decreases were observed in VV, VT, ICI, BC and VTNVC. Treatment with 13-*cis*-RA did not lead to any changes in PVR, VE, BCD or RT. Intravenous administration of duloxetine to animals previously treated with 13-*cis*-RA led to decreases in DOI, ANVC and FNVC, and to increases in BC and VTNVC. After administration of duloxetine, no statistically significant changes were observed in BP, TP, MVP, PVR, VT, VE, ICI, BCD or RT (Figs. 1, 2 and 3).

### Behavioural study

**Fig. 4:**
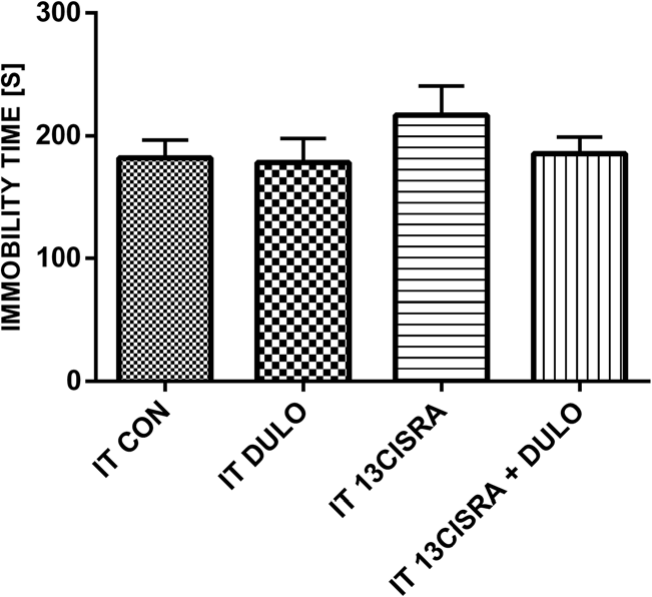
Immobility times in rats in the different treatment groups. All results are presented as means ± SEM (*n* = 15 rats per group). The data obtained were evaluated using one-way analysis of variance (ANOVA) followed by Tukey’s post hoc test

**Fig. 5:**
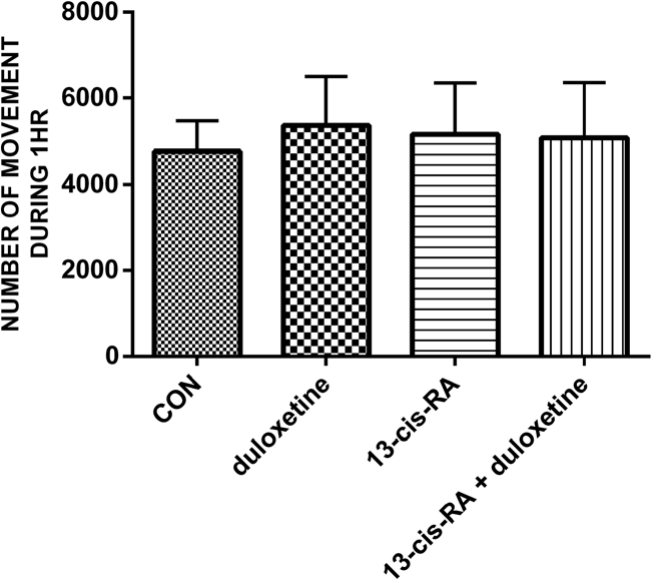
locomotor activity (number of movements) in rats in the different treatment groups. All results are presented as the means ± SEM (*n* = 15 rats per group). The data obtained were evaluated using one-way analysis of variance followed by Tukey’s post hoc test

Duloxetine administered as a single dose did not produce any statistically significant changes in the animals in the forced swim test. Treatment with 13-*cis*-RA led to an increase in the immobility time compared with the control group. Treatment with duloxetine in animals previously treated with 13-*cis*-RA led to a significant decrease in the immobility time to the level found in the control animals (Fig. 4). None of the treatments examined affected the locomotor activity of the animals (Fig. 5).

### Body weight

No statistically significant differences in body weight were seen among the treatment groups. The mean body weights of the animals in the control group and treatment groups were 71.3 ± 4.5 g and 76.1 ± 6.1 g, respectively, at the beginning of the experiments, and 242.6 ± 12.4 g and 231.7 ± 10.6 g, respectively, at the end of the experiments.

### 13-*cis*-Retinoic acid plasma levels

The level of 13-*cis*-RA in the plasma from animals treated with 13-*cis*-RA was 0.58 ± 0.021 μg/ml, while the plasma from animals in the control group contained essentially no measurable 13-*cis*-RA.

### CRF levels

**Fig. 6:**
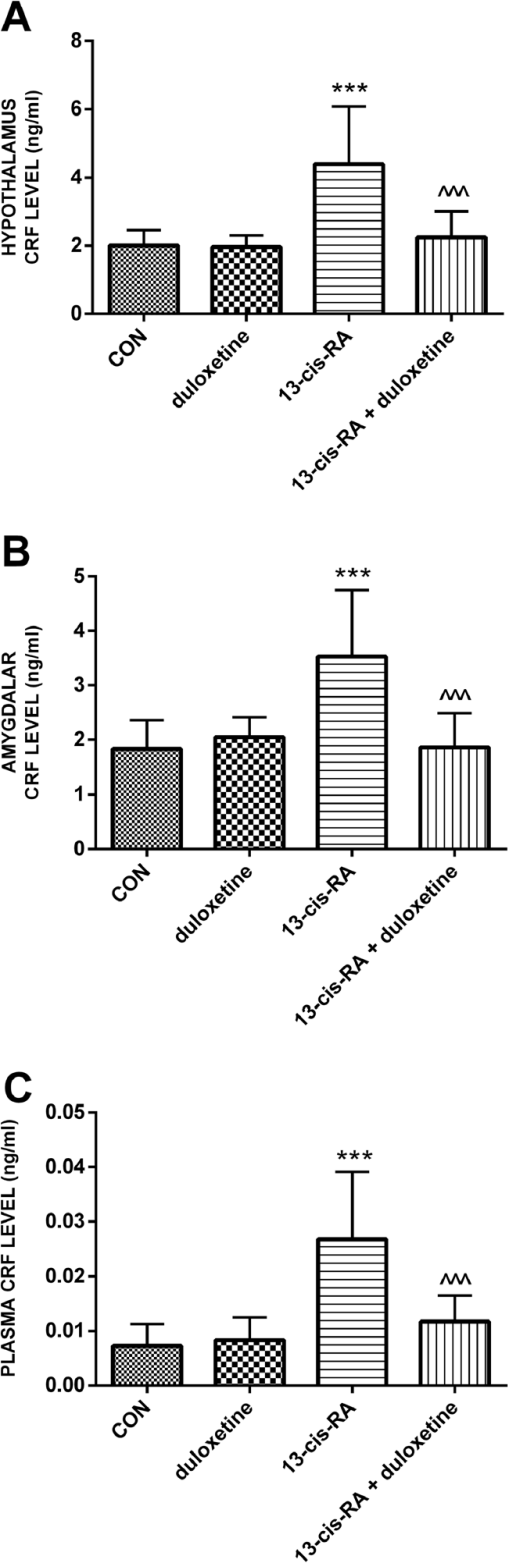
Mean CRF levels in the hypothalamus, amygdala and plasma of rats in the different treatment groups (*CON* control group). **a** Hypothalamus (***p < 0.001, CON vs. 13-*cis*-RA; ^^^*p* < 0.001, 13-*cis*-RA vs. 13-*cis*-RA + duloxetine). **b** Amygdala (***p < 0.001, CON vs. 13-*cis*-RA; ^^^*p* < 0.001, 13-*cis*-RA vs. 13-*cis*-RA + duloxetine). **c** Plasma (***p < 0.001, CON vs. 13-*cis*-RA; ^^^*p* < 0.001, 13-*cis*-RA vs. 13-*cis*-RA + duloxetine). All results are presented as means ± SEM (*n* = 15 rats per group). The data obtained were evaluated using one-way analysis of variance followed by Tukey’s post hoc test. ^^^*p* < 0.001, ***p < 0.001

Treatment with a single dose of duloxetine had a statistically significant impact on CRF levels in both the examined brain structures and the plasma. The animals treated with 13-*cis*-RA showed increased levels of CRF in he hypothalamus, amygdala and plasma. Administration of duloxetine to rats previously treated with 13-*cis*-RA caused CRF levels to decrease in the hypothalamus, amygdala and plasma (Fig. 6).

## Discussion

The model of depression induced by 13-*cis*-RA, which has also been shown to cause DO symptoms, was used in this study. Clinical trials have shown that therapeutic doses of 13-*cis*-RA administered to patients with severe acne can induce depressive behaviour [21]. These findings were confirmed in preclinical studies in which long-term administration of 13-*cis*-RA led to increases in immobility time in the forced swim test, which is consistent with increased depression-related behaviour [22]. In the experiments presented here, the rats received a dose identical to that used in acne treatment, and the determined concentrations of 13-*cis*-RA in plasma were analogous to those found in humans [23].

Treatment with duloxetine was found to have an antidepressant effect, as shown by significant decreases in the immobility time to the times observed in the control animals, with a simultaneous lack of effect on their locomotor activity. Treatment with duloxetine led to significant decreases in DOI, ANVC and FNVC and to increases in BC and VTNVC. Duloxetine appeared to have no effects on the other analysed parameters, namely BP, TP, MVP, PVR, VT, VE, ICI, BCD and RT. The demonstrated decrease in DOI seems to be very important, considering that an increase in this parameter is a characteristic feature of DO that allows its intensity to be determined. It is believed that DOI reflects the contraction activity of the bladder with far greater precision than ANVC, FNVC, MVP, ICI, BP or BC [18, 19]. The observed increase in VTNVC following administration of duloxetine is very interesting, because this parameter is considered a preclinical equivalent to the volume at the first involuntary detrusor contraction measured during urodynamic investigations in humans [24]. The dose used in this study was the minimally effective dose, which showed antidepressant activity in the forced swim test and simultaneously affected the cystometric parameters DOI, ANVC, FNVC, BC and VTNVC. This might suggest that duloxetine could be used in the pharmacotherapy of OAB dry, especially in patients with accompanying depression. It cannot be ruled out that higher doses administered for a longer period could effectively treat OAB wet.

Recently published studies on the link between OAB and depression were population-based epidemiological surveys and yielded conflicting results [4, 25]. In a study by Lai et al. [26], 27.5% of OAB patients had depression as confirmed by the Hospital Anxiety and Depression Scale (HADS ≥8), and affected individuals reported significantly higher HADS-D depression scores than age-matched controls. No standards for the treatment of patients with DO and accompanying depression have yet been developed, because OAB is a syndrome and does not exist as an entity. So far only one case report has been published in which the authors confirmed the efficacy of duloxetine in a female adolescent with OAB and depression [14]. It is worth noting that the patient showed resistance to antimuscarinic drugs and standard antidepressants.

The rationale for the use of duloxetine in DO stems from the direct inhibition of sensory afferents by the serotonergic system, which results in a reduction in bladder detrusor activity and a modulating effect of serotonin and noradrenaline on the micturition centres in the sacral spinal cord [27]. It has been shown that acute administration of antidepressants increases the threshold for the spinal voiding reflex, while chronic administration affects the CNS-related components of the micturition reflex, which suggests that duloxetine could inhibit bladder overactivity in patients with coexisting depression [15]. There are reports that SNRIs can induce urine retention, but this was not confirmed in our study. Duloxetine was found to have no effect on PVR.

It ha been demonstrated that duloxetine increases bladder capacity and inhibits DO induced by acetic acid, and its effects are reversed by administration of a nonselective 5-HT receptor antagonist [28]. The fact that DO induced by chemical irritation is mediated at least in part by a spinal reflex activated by nociceptive C-fibre afferents of the urinary bladder suggests the possibility that duloxetine affects not only supraspinal micturition control mechanisms but also spinal reflexes.

Clinical trials have shown that duloxetine reduces the number of frequency and urgency episodes, and improves quality of life scores, while not changing the mean voided volume [15, 16]. It has not been confirmed that it has any effects on heart rate, blood pressure or arrhythmogenic tendencies on EEG, which is significant because the majority of patients with depression and OAB are elderly. Duloxetine was found to have greater efficacy than placebo in patients with OAB due to either DO or sensory urgency, and also in patients with MUI [16, 29]. Due to the drug’s clinically proven effectiveness in the treatment of depression, it has been suggested that it should be the first-choice treatment in patients with depression and accompanying OAB [15].

In this study duloxetine was found to lower CRF levels in the hypothalamus, amygdala and plasma that were previously increased by administration of 13-*cis*-RA. CRF hypersecretion seems to be a common element in the aetiopathogenesis of depression and OAB stemming from disturbances to the hypothalamic–pituitary–adrenal axis. There is growing evidence to support the considerable impact of CRF on urinary bladder function. This neurohormone has been found to decrease the threshold of afferent impulsation of the micturition reflex and to increase bladder detrusor contraction activity. CRF also plays an important role in micturition control mechanisms at the level of the spinal cord [30]. Preclinical studies have shown that selective CRF_1_ antagonists reduce DO symptoms [10]. It has been shown that selective destruction of afferent C-fibres leads to decreased expression of CRF, which indicates that this neurohormone is important for conducting the afferent impulsation from the urinary bladder [31]. Increased CRF expression has also been found in the urothelium and suburothelial ganglions in an animal model of cystitis.

It has been shown that brain regions characterized by a high density of CRF_1_ receptors (such as the hippocampus, the prefrontal cortex and Barrington’s nucleus in the brainstem amygdala), apart from their well-known role in the body’s response to stress factors, are also involved in regulation of the micturition reflex, and this suggests that antagonists to this receptor might be an interesting target for OAB pharmacotherapy, especially in patients with accompanying depression [32]. Another CNS structure found to be a common element in the aetiopathogenesis of OAB and depression is the anterior cingulate cortex, whose increased activation has been detected on MRI in patients with OAB and patients with depression. SPECT also shows features of hypoperfusion and hypometabolism of this structure in both these groups of patients.

### Conclusions

This study provides further data concerning the links between the aetiopathogenesis of OAB and depression. Three main findings concerning the action of duloxetine should be particularly underlined: (1) it reduces the cystometric changes of DO induced by 13-*cis*-RA administration, (2) it shows antidepressant activity, and (3) it decreases CRF levels in the hypothalamus, amygdala and plasma. This is the first study showing the efficacy of duloxetine in an animal model of DO induced by depression. Further studies in patients with DO and coexisting depression are warranted to confirm these experimental results.
